# Nuclear Tau and Its Potential Role in Alzheimer’s Disease

**DOI:** 10.3390/biom6010009

**Published:** 2016-01-07

**Authors:** Mahmoud Bukar Maina, Youssra K. Al-Hilaly, Louise C. Serpell

**Affiliations:** 1 School of Life Sciences, University of Sussex, Falmer, Brighton BN1 9QG, East Sussex, UK; M.Bukar-Maina@sussex.ac.uk (M.B.M.); ya45@sussex.ac.uk (Y.A.H.); 2 Department of Human Anatomy, College of Medical Science, Gombe State University, Gombe 760, Nigeria; 3 Chemistry Department, College of Sciences, Al-Mustansiriyah University, Baghdad, Iraq

**Keywords:** tau, nucleus, nucleolus, Alzheimer’s disease, paired helical filament, neurofibrillary tangles

## Abstract

Tau protein, found in both neuronal and non-neuronal cells, forms aggregates in neurons that constitutes one of the hallmarks of Alzheimer’s disease (AD). For nearly four decades, research efforts have focused more on tau’s role in physiology and pathology in the context of the microtubules, even though, for over three decades, tau has been localised in the nucleus and the nucleolus. Its nuclear and nucleolar localisation had stimulated many questions regarding its role in these compartments. Data from cell culture, mouse brain, and the human brain suggests that nuclear tau could be essential for genome defense against cellular distress. However, its nature of translocation to the nucleus, its nuclear conformation and interaction with the DNA and other nuclear proteins highly suggest it could play multiple roles in the nucleus. To find efficient tau-based therapies, there is a need to understand more about the functional relevance of the varied cellular distribution of tau, identify whether specific tau transcripts or isoforms could predict tau’s localisation and function and how they are altered in diseases like AD. Here, we explore the cellular distribution of tau, its nuclear localisation and function and its possible involvement in neurodegeneration.

## 1. Introduction

Tau (tubulin-associated unit) is a low molecular weight protein, first identified by Weingarten *et al.* which showed the capacity to promote microtubule assembly *in vitro* [1]. It is expressed in higher eukaryotes and found in both neuronal and non-neuronal cells, but predominating in neurons [2,3,4,5]. Besides its widely known role in microtubule assembly and stability, tau has become known for many other functions, such as the maintenance of axonal transport and providing linkage for signal transduction [5,6,7,8]. Tau is a product of the microtubule-associated protein (MAPT) gene, located on chromosome 17q21.1 [9,10,11]. The tau gene, through complex post-transcriptional processing, yields predominantly three transcripts: a less abundant 2kb tau transcript which encodes for a tau mainly targetted to the nucleus [12]; 6kb transcript which encodes for tau predominantly directed to the soma/axons in the central nervous system (CNS) [11,13]; and 8/9 kb transcript producing a tau preferentially expressed in the retina and peripheral nervous system (PNS) and with apparent molecular weight of about 110–120 kDa, often called high molecular weight (HMW) tau [14,15]. The 8/9 kb transcript arises from the inclusion of exon 4A from the tau gene during tau pre-mRNA processing. The 2 kb and 6kb transcripts result from the same pre-mRNA polyadenylated on different sites, with the 2 kb transcript having poly-A tail addition about 3.5 kb before that of the 6 kb transcript. This is responsible for the different preferential location of their products, and may impact on their function and stability [15,16,17,18].

**Figure 1:**
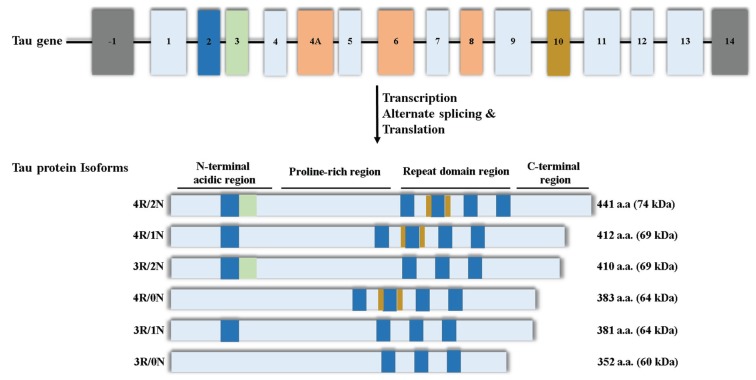
Tau gene, protein isoforms, and structure. The tau gene has 16 exons; exon 1, 4, 5, 7, 9, 11, 12 and 13 (**light blue**) are constitutively transcribed in the CNS [5]. Exon 4A, 6 and 8 (**orange**) are rarely expressed in the brain but included in mRNA of most peripheral tissues, while exon 14 forms part of the 3ʹ untranslated region of the tau mRNA [11,21]. Alternate splicing of exon 2 (**blue**), 3 (**Green**) and 10 (**Yellow**) in the CNS generates the widely known six isoforms of tau; 352–441 amino acids in length and 60–74 kDa in weight on SDS-PAGE [5]. Depending on the inclusion and/or exclusion of exon 2, 3 and 10, tau can have three or four (3R/4R) microtubule binding repeats and the presence or absence of 1 or 2 N-terminal inserts, leading to the six widely known isoforms of tau in the CNS. Structurally, the tau molecule is subdivided into four regions; an N-terminal acidic region; Proline-rich region/domain (PRD), repeat domain region and a C-terminal region.

The tau gene has 16 exons, of which, exon 2, 3, 4A, 6, 8, 10 and 14 are alternatively spliced [10,11]. Theoretically, splicing of this gene could yield up to 30 different variants of tau protein, thus creating an additional layer of complexity to the distribution of tau in different tissues [6,11,15,19,20]. The alternate splicing of exon 2, 3 and 10 generates the six widely known isoforms of tau in the CNS, ranging from 352 to 441 amino acids in length and 60–74 kDa in weight on SDS-PAGE (Figure 1) [5,6]. The smallest isoform is found in the foetal brain, expressing three microtubule-binding repeats on its C-terminal (3R) and 0 N-terminal inserts, and is called foetal tau. The other five isoforms are bigger and predominantly found in the adult brain, having either three or four (3R/4R) microtubule binding repeats and the presence or absence of 1 or 2 N-terminal inserts [6].

Structurally, tau is subdivided into four regions; an N-terminal acidic region; a Proline-rich domain (PRD), repeat domain region and a C-terminal region, and the epitopes across these regions vary depending on the tau isoform [5,6]. Isoform localisation preference also exists between developmental stages, tissues, cell lines, brain regions and intracellular compartments [12,15,19,20,22]. For instance, in the murine brain, the 0 N and 1 N tau predominate in the cell body/axons and nucleus, respectively [13]; and in SH-SY5Y neuroblastoma cells, high and low molecular weight tau both exist, and tau may predominantly localise to the nucleus or cytoplasm depending on whether the cells are differentiated or not [23].

It is thus clear that the complex posttranscriptional processing of the tau message yields numerous isoforms with varying localisation. However, tau researchers have placed more focus on the tau localised to the axons, likely due to its well-known role in microtubule stability and dynamics, axonal transport and involvement in the pathogenesis of many neurodegenerative diseases, such as Alzheimer’s disease (AD). In AD, tau misfolds to form paired helical filaments (PHF) which are deposited in neurofibrillary tangles (NFTs) [5], which together with amyloid plaques, are the principal hallmarks of the disease as described by Alois Alzheimer’s in 1906 [24].

**Figure 2:**
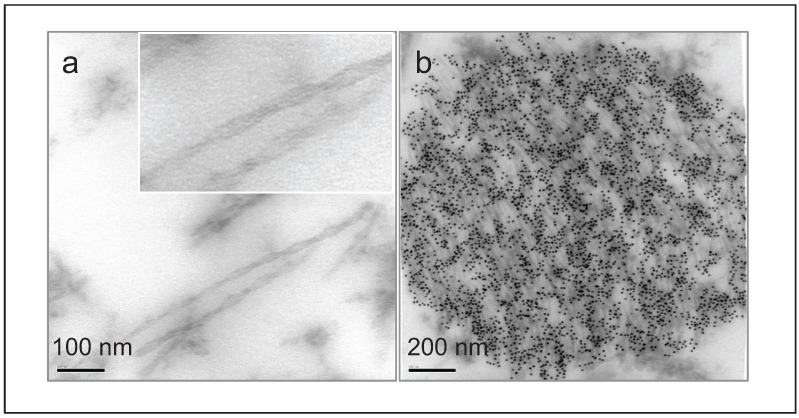
Transmission electron micrographs showing (**a**) paired helical filaments in human AD brain; and (**b**) a neurofibrillary tangle, immunogold labelled with anti-tau antibody.

Electron microscopy reveals that PHFs (Figure 2b) are made up of a twisted ribbon-like structure (Figure 2a), whereby two filament twist around one another [25]. Structurally, both tau filaments from human brain and from *in vitro* assembly of recombinant tau protein have cross-β structure [26]. However, the exact mechanism of tau assembly into PHFs is still not well understood. Both hyperphosphorylation and truncation have been proposed as key molecular events in the abnormal tau aggregation leading to the formation of PHFs. While much attention has focused on hyperphosphorylation as a mechanism to induce the self-assembly of tau, an alternative is that tau truncation is the trigger [27,28,29,30].

Many studies have proposed that tau phosphorylation is associated with tau assembly into PHFs [27,28]. However, these studies have been based upon *in vitro* experiments and animal models, and so it has been argued that further supporting evidence is required using human AD samples. It has been shown that the core of PHFs contains truncated forms of tau protein [31]. Novak *et al.* demonstrated that truncation is mediated by specific cleavage events *in vivo* [29,32]. Furthermore, it has been suggested that truncated tau can serve as a nucleus for the assembly of endogenous tau into neurofibrillary tangles [33]. To understand and gain more insight into the relationship between phosphorylation and truncation, a more recent immunohistochemical study of AD brain tissue revealed that truncated tau represents an early neurotoxic form and proposed that phosphorylation may play a neuroprotective role by inhibition of tau aggregation [34].

Regardless of the mechanism involved in tau aggregation, many studies have revealed the localisation of tau in multiple cellular compartments (discussed later). This has led to the suggestion that tau is a multifunctional protein, as such; its involvement in neuronal physiology and pathology needs to be regularly reviewed in light of new discoveries. Here, we discuss the unconventional localisation and function of tau, especially, as it relates to the nucleus, and how it may play a role in neurodegenerative diseases like AD.

## 2. Nuclear Tau and Alzheimer’s Disease

### 2.1. Cellular Localisation of Tau

Tau is a cytosol-enriched protein, distributed within the somatodendritic compartment, but predominating in the axons [35,36], that regulate microtubule assembly and stability, and axonal transport of vesicles and organelles [7,37]. However, numerous studies have identified it in different subcellular compartments. Its localisation to the microtubules of growth cones [38,39] and mitotic spindle [40] has raised questions as to whether it has a non-microtubule polymerizing function, since the microtubules in these two locations are more dynamic than axonal microtubules [41]. Tau, mostly in a dephosphorylated state, has been localised in the plasma membrane of different cell lines [42]. Its N-terminal domain mediates the interaction with the plasma membrane [6]. In rat cortical neurons, a significant quantity of tau is localised to the membrane and this tau is dephosphorylated at Tau-1, AT8 and PHF-1 epitopes [43]. This tau-membrane interaction is highly dynamic and depends on phosphorylation, such that inhibiting Casein kinase 1 (CK1) or *Glycogen synthase kinase 3*β (GSK3β) significantly increased tau translocation to the membrane, and mimicking tau N-terminal phosphorylation prevented the tau-membrane localisation [43]. Tau has also been identified in the lipid rafts of the Tg2576 mouse brain, the AD brain [44], and lipid rafts of primary neurons, where it is regulated by Aβ oligomers [45]. In neurons, it localises in a good quantity within the synapses [46]. This different localisation of tau provides evidence for its role in non-microtubule-associated functions, such as signal transduction [6,41]. In support of this, Ittner *et al.* [8] showed that tau localised to the dendrites mediates Aβ toxicity by targeting the sarcoma (Src) kinas FYN to post-synaptic NMDA receptors in the mouse brain. Hyperphosphorylation of tau in AD models has also been shown to mislocalise tau to the dendrites, where it alters synaptic function by affecting glutamate receptor trafficking [47]. These studies collectively suggest that tau-associated factors that promote neurodegeneration, such as tau phosphorylation, could change the localisation of tau and its functions, and trigger its somatodendritic accumulation, axonal microtubule disassembly, PHF formation and neurodegeneration [48].

Apart from the compartments above, tau has been localised to the ribosomes of both neurons and astrocytes in the AD brain [35,49]. Papasozomenos and Su also found abnormally phosphorylated tau associated with purified ribosomes from AD brains but not from control brains [50]. Its association with the ribosomes raised interesting questions on whether it plays any function related to protein synthesis. To date, the functional relevance of this association is still elusive. In the human brain, tau has also been found to partially colocalise with KDEL (a marker of the endoplasmic reticulum), and Golgin 97 (a marker of Golgi apparatus), and that this colocolisation was more apparent in the AD brain [51]. In the AD brain, tau also colocalised with the marker of the mitochondria (COX IV) [51]. Tang *et al.* [51] showed that cells engineered to have upregulated mTor activity also showed tau signals with the endoplasmic reticulum, golgi, and the mitochondria, generally indicating that the enhanced mTor activity caused the trafficking of tau and its subsequent release into the extracellular space. Aside from these extra-nuclear locations of tau, it has been localised within the nucleus of the mouse brain neurons [52,53], within the nucleus and nucleolus of different undifferentiated primate cell lines [3], and the nucleus of the AD and control brain [50]. The evidence reviewed so far demonstrate that tau is a ubiquitous protein, highly dynamic, with a broad range of potential functions and whose functions and localisation are altered in neurodegenerative disease. Considering the importance of the nucleus in cellular homeostasis, out of all the different localisations of tau, we are particularly interested in its nuclear localisation as this raises many questions regarding its likely role in the nucleus, and how this is influenced in tauopathies. Hence, this review focuses on nuclear tau and its potential nuclear roles in physiology and pathology.

### 2.2. Nuclear Tau: Three Decades of Discovery

In 1988, Metuzals and coworkers published a paper demonstrating the presence of PHF profiles within the nucleus of AD brain biopsies [54]. Around the same time, using immunofluorescence microscopy with Tau-1 antibody (which detects dephosphorylated tau), tau was localised within the nucleus of CG human neuroblastoma cells, specifically at the nucleolar organiser region (NOR) of the acrocentric chromosomes in dividing cells—the site of rRNA genes; and the fibrillar region of the nucleolus in the interphase cells—the site of rRNA transcription [3]. Similar tau staining patterns were found in other primate cell lines, but no tau immunoreactivity was observed in the non-primate cell lines used in their study. This finding was further confirmed by the same group with immunoblotting using the mAb Tau 46.1 [12], and sense and antisense transfection strategies [55] in neuroblastoma cells. Similarly, the neuroblastoma LA-N-5 cell line also showed nucleolar tau localisation [56] and Thurston *et al.* [57] revealed that other human non-neuronal cells like fibroblasts and lymphocytes contain nucleolar tau. This discovery of nuclear tau was very significant, as it provided strong evidence for a potential non-microtubule associated function for tau at that time. Accordingly, Lu and Wood [58] showed that the microinjection of fluorescently-tagged bovine tau to cultured Chinese hamster ovary (CHO) cells (which do not normally express tau protein) showed the accumulation of tau within both the nucleolus and the centrosome. Cross *et al.* [59] colocalised tau with the centrosome of interphase cells of the Huh-7 cells, SW-13 cells, and normal human fibroblasts. Using *in vitro* assays, they also showed that the centrosomal tau promotes microtubule assembly at the centrosomes [59].

Subsequently, nuclear tau was localised in non-primate cell lines; such as the rat brain cell line B103 [60], cultured mouse cortical neurons [52], and the mouse brain [13,53]. All this evidence makes a strong case that tau is a bonafide nuclear protein, and also suggests that although nuclear tau could be a shared phenomenon between different cell types of different species, some cells, especially primate cells, show prominent nucleolar tau localisation [3,61,62]. Considering that nucleolar tau was found predominantly in dividing cells, it was assumed that it functions only in dividing cells, such that its expression ceases after differentiation [12]. However, the same group later identified nuclear tau in the normal and AD brain with occasional scanty nucleolar staining [50]. The tau staining from their study is mostly extranucleolar, prompting the authors to suggest that tau may have a nuclear function in postmitotic neurons, and the absence of nucleolar tau may indicate its function is no longer necessary in terminally differentiated cells. We were thus surprised and excited to localise nucleolar tau in retinoic acid and brain-derived neurotrophic factor (BDNF) differentiated SHSY5Y cells [63]—a widely used model for studying cellular changes associated with tauopathy. We used different antibodies to confirm its localisation, and also colocalised it with various nucleolar proteins using both confocal and immunogold transmission electron microscopy (manuscript in preparation). Using immunogold labeling, we have also localised an appreciable amount of nucleolar tau in the normal and AD brain, indicating that it is also a bonafide nucleolar protein (manuscript in preparation). The difficulty in visualizing nucleolar tau in the work of Brady *et al.* [50] could be attributed to the processing protocol, which affects the visualisation of tau [64].

We are still far from understanding the complete function of tau, especially as it relates its role in the nucleus. This is partly complicated by the presence of many isoforms of tau in primate and non-primate cells and the human brain. Indeed, the identity of nuclear tau, such as its posttranslational modifications and the isoforms that exist within the nucleus is largely elusive. Post-translational modifications of tau are important disease modifiers in tauopathies [5], and the ratio of the different isoforms of tau also changes in some tauopathies [21]. To appreciate the role of nuclear tau in physiology and pathology, there is the need to understand the real identity of the nuclear tau.

### 2.3. The Identity of Nuclear Tau

Alternate polyadenylation and alternative splicing could generate over 30 variants of tau. Most of our knowledge has focused mainly on the 6 kb transcript of tau and the six widely known isoforms in the central nervous system (CNS) (Figure 1). Whether nuclear tau is generated from a distinct transcript and/or whether specific nuclear isoforms exist is not clear. It is possible that the tau that localises to the microtubules is different from the one that localises to the nucleus. Otherwise, localisation of tau with microtubule-binding message/function would render the microtubule undersupported and vulnerable. To begin to tackle this question, Liu *et al*. observed that 1N4R tau isoform in the murine brain localises mainly to the nucleus, with some quantity in the soma, and dendrites, but not the axons [13]. Interestingly, Wang *et al.* previously showed that the 2 kb tau transcript produces mainly nuclear tau, with a small amount of cytosolic tau showing non-microtubule binding function [12]. Whether the murine brain also contains a 2 kb tau transcript is not known, but it would be interesting to investigate if there is a special targeting at the transcript level that specifies the distribution of the 1N4R message to the nucleus. Other isoforms (e.g., 2N4R) and products of the 6 kb tau transcript can also localise in small quantities within the nucleus [12,13]. Decoding the transcripts targeting and isoform localisation of nuclear tau would enable a detailed understanding of tauopathies, some of which arise from altered splicing and balance of tau isoforms [21].

Apart from the post-transcriptional processing that yields different tau transcripts and isoforms, tau undergoes many post-translational modifications, such as phosphorylation [5]. Tau phosphorylation has been suggested to be an important modifier of its function and the induction of toxicity in neurons [43,65]. It is, therefore, important to investigate whether phosphorylated tau can also localise to the nucleus and the functional relevance of this localisation. Indeed, Greenwood and Johnson [56] found that nuclear tau in LAN-5 neuroblastoma cells may exist in both phosphorylated and native state, to a similar extent to the tau pool in the cytoplasm. *In vitro* phosphorylation of an intact isolated nucleus incubated with ATP Gamma ^32^P revealed that the nuclear tau is likely not to be phosphorylated in the nucleus, but rather in the cytoplasm before translocation into the nucleus. Other studies confirmed that a quantity of nuclear tau could exist in a phosphorylated state in normal cell lines, mouse brain and the human brain [20,50,60,66]. However, some studies showed that tau, especially nucleolar tau, can only be detected with Tau 1 antibody, which recognises tau dephosphorylated at serine 195, 198, 199 and 202 [3,12,57]. Tau in the mouse brain also exists mainly in a dephosphorylated state [64], especially under stress conditions [52]. Nuclear tau in the human brain can also exist in a non-phosphorylated state [50]. Generally, these findings appear to suggest the existence of both phosphorylated and dephosphorylated nuclear tau, which may vary depending on cell type and intranuclear localisation, with the nucleolus restricted to mostly non-phosphorylated tau. So far, it is not entirely clear what functional role is played by the nuclear phosphorylated and non-phosphorylated tau. The nuclear compartment could also harbour an abnormal tau or tau on the path of transformation to a pathological state. Using both Pr5 mice that express the P301L tau mutation, and ΔTau74, which express truncated form of wild-type human tau with no microtubule-binding domain, Lu *et al.* showed that tau accumulates in the nucleus [64]. Further, the first paper that hinted the localisation of tau to the nucleus, identified PHF strands within the AD brain nucleus [54]. Tau rod-like deposits with ordered filamentous ultrastructure were also localised within the brain, in nuclei, of subjects with AD and Huntington disease [67]. These finding collectively suggest a role for tau within the nucleus in normal and disease conditions.

We generally have a modest understanding of the functional role of tau isoforms, but the work of Wang *et al.* [12] and Liu *et al.* [13] strongly suggests that the bulk of nuclear tau may arise from a distinct transcript and comprised of a dominant isoform. It is crucial to decipher whether this nuclear tau typically interacts with and influences the DNA, and if/how this is important in pathology.

### 2.4. Capacity of Tau to Interact with the DNA

As far back as 1975, Bryan *et al.* showed that RNA could inhibit microtubule assembly *in vitro*, through the reduction of the activity of a protein essential for tubulin assembly [68]. They showed that tau protein could serve as this protein whose activity is reduced by the RNA. This provided a preliminary evidence for interaction between tau and RNA. Indeed, two decades later, it was further shown that RNA could induce the aggregation of tau into AD-like PHFs [69]. Considering the relationship between the DNA and RNA, this finding also suggested a possible interaction between tau and the DNA. Indeed, Corces *et al.* showed that brain depolymerised microtubule-associated proteins bind the DNA with high affinity [70]. Using an *in vitro* assay, Corces *et al.* further showed that DNA inhibits microtubule assembly in a concentration-dependent manner, indicating that microtubule-associated proteins have more affinity to the DNA than to the microtubules [71]. In the study, they explicitly showed that tau protein-containing microtubule fractions bind the DNA. Hua and He later found that addition of native tau to a solution of Calf Thymus DNA (CTDNA) increased the melting temperature (T_m_) of the DNA from 67 °C to 81 °C in a concentration-dependent manner [72]. Similarly, tau protected pBluescript-II SK DNA from denaturation by raising its T_m_ from 75 °C to 85 °C. Kinetics study of tau and DNA further showed that tau can stabilise double-stranded DNA [72]. This study made a strong case for tau as a DNA binding protein *in vitro*. However, it is unclear whether the DNA interacts only with native tau or whether it can interact with tau modified by posttranslational modifications, such as phosphorylation. Hence, the same group looked at the interaction of phosphorylated or aggregated tau with the DNA. Using an *in vitro* approach, they showed that tau phosphorylated by a neuronal cdc2-like kinase (NCLK) retains its ability to bind the DNA and also increases the CTDNA melting T_m_ [72]. Interestingly, Hua and He observed that when the conformation of tau is changed by aggregation, electrophoretic mobility shift assay (EMSA) and agarose gel retardation assay both showed that phosphorylated and native tau lose the ability to bind the DNA [72]. However, a recent study to further characterise the nature of this interaction revealed strongly reduced or loss of capability of tau phosphorylated by GSK-3β for binding and protecting the DNA against thermal denaturation [73]. Although both NCLK and GSK-3β phosphorylate tau on multiple epitopes, including the PHF epitopes, GSK-3β can phosphorylate tau on additional epitopes not phosphorylated by NCLK and has been suggested to play a dominant role in tau phosphorylation [74]. Hence, this could be the reason for the discrepancy between the two studies on phosphorylated tau-DNA interaction [73,75]. Using other approaches, other studies also showed a similar reduction of the phosphorylated tau-DNA interactions [76,77]. Generally, these findings revealed significant information about the interaction of tau with the DNA which will contribute to understanding the role of nuclear tau in AD [5]. Based on these findings, it was postulated that dysfunction in the phosphorylation of tau, such as in AD, might lead to its aggregation, thereby affecting its ability to both stabilise the microtubule and protect the DNA [78]. In order to further characterise the nature of the tau-DNA interaction, Hua *et al.* investigated the binding of tau to double-stranded (dsDNA) or single-stranded (ssDNA) DNA, and the nature and flexibility of this binding. Their results revealed that tau binds to dsDNA, but not ssDNA, and that this binding is rapid, dynamic and reversible and occurs in a cooperative, rather than sequence-specific fashion [78]. It was suggested that the binding probably occurs via a charge effect since incubation of tau-DNA solution in an increasing concentration of a high ionic strength buffer (NaCl) in the plasmid incubation medium led to a NaCl concentration-dependent binding of tau to the DNA. Electron microscopy showed that tau clustered around the DNA like a necklace. In contradiction to Hua *et al.* [78], a study by Krylova *et al.* [79], using kinetic capillary electrophoresis (KCE), found that tau could not only bind dsDNA, but it can also bind a ssDNA in sequence-specific fashion, and that it induces the denaturing of dsDNA by binding to one of its strands sequence specifically. Hence, in an effort to clearly elucidate the interaction of tau with the DNA and therefore understand the functional significance of this interaction, Qu *et al.* using atomic force microscopy (AFM) showed that monomeric tau molecules bind the DNA at a molar ratio of about 1:10 (tau/DNA), the equivalent to about 1 tau molecule to 700 bp of dsDNA [80]. Further studies revealed that tau binds and bends the DNA through AT-rich minor groove of the DNA, likely through an electrostatic interaction with the ε-amino group of its lysine residues on its PRD and microtubule binding domain (MBD); and that tau preferentially binds DNA sequences of about 13 bp or longer [81]. However, a recent study using nuclear magnetic resonance spectroscopy (NMR) further revealed that this interaction may be through the second half of the PRD of tau (R209 to A246), and a second interaction site on its MBD in the R2 repeat region (K267 to S289) [77]. The authors also showed that tau interacts with not only AT-rich regions but GC-rich oligonucleotides, indicating a general binding with the DNA backbone, independent of the bases, although hydrophobicity has also been suggested to be essential in the tau-DNA interaction [76]. Therefore, there is sufficient evidence to indicate the capacity of tau to interact, stabilise and bend the DNA. However, most of these experiments showing tau-DNA interaction were conducted outside a cellular environment, mostly using recombinant proteins.

Greenwood and Johnson earlier found that out of the total tau in L-A-N-5 neuroblastoma cells, 14% are localised within chromatin fraction containing DNA, chromatin, and associated proteins [56]. This confirmed that tau could form a complex with the DNA within a cellular environment. Moreover, they further showed that the tau in the chromatin fraction could exist in a phosphorylated state, supporting the work of Hua and He which showed the capacity of phosphorylated tau to interact with the DNA [75]. Using immunofluorescence microscopy, Sjoberg *et al.* provided evidence *in situ* of tau-DNA interaction [62]. They showed that tau colocalised with diMeK9H3 and centromeric α satellite DNA in human fibroblasts. They further showed that tau also binds to human α satellite DNA sequences and murine γ-satellite DNA sequences. Rossi *et al.* also showed that tau localises to the spindle poles and the mid-body in dividing cells [2]. Sultan *et al.* using immunoprecipitation in primary neurons further showed that tau interacts *in situ* with the DNA [52]. Using netropsin—a polyamide that binds the minor groove of dsDNA through AT-rich sequences; and methyl green—a major groove binding drug—Sultan *et al.* confirmed that tau binds the minor groove of the DNA. All these studies indicate that tau is a DNA-binding protein, so what is the functional role of nuclear tau?

### 2.5. Functional Role of Nuclear Tau

**Figure 3:**
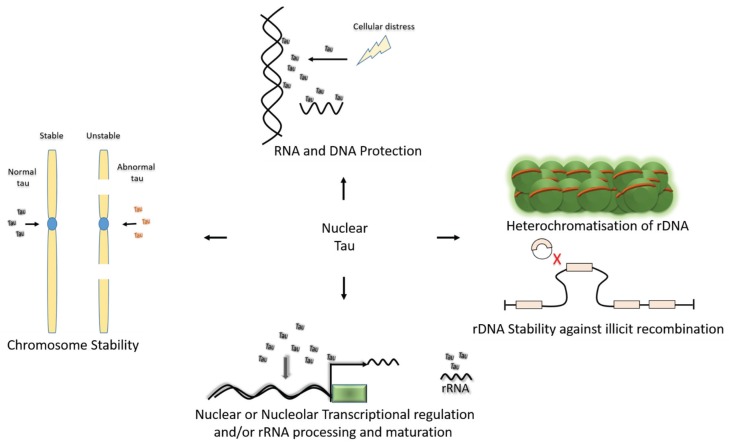
Potential functions of Nuclear tau. Tau has been shown to protect the DNA and RNA from cellular distress [52,53]. It has been localised within the nucleolus, at the vicinity of the rDNA and associated with a markers of the heterochromatin, within the nucleolus, it specifically localises to the dense fibrillar component (DFC) of the nucleolus—a region involved with rDNA transcription and processing of nascent pre-rRNA; collectively, this suggests a potential role for tau in either rDNA heterochromatisation and stability, rDNA transcription and/or rRNA processing and maturation [3,57,62]. Data from cell culture and human cells with varying tau mutations also provides strong evidence for tau in the maitenance of chromosomal stability [2,87]. The nature of interaction of tau-DNA interaction also suggests it could be be involved in nuclear transcriptional regulation (See below for more detailed discussion). All these functions need further research to be completely validated.

Tau’s localisation within the nucleus is particularly interesting, considering the importance of the nucleus in cellular processes (Figure 3). Looking at cell lines and the human brain, nuclear tau exists in different isoforms, with some cells showing diffuse nuclear staining of tau and some showing major nucleolar tau signal [3,12,13,50,59,64]. While discussing the role of nuclear tau, it may be important therefore to make a distinction between the nuclear tau ubiquitously distributed within the nucleus and tau predominantly localised to the nucleolus. The difference in their nuclear distribution could assign different roles for them in neuronal physiology and pathology. The localisation of tau to the nucleolus in interphase cells or the NOR of dividing cells is very intriguing considering the role of the nucleolus in cellular life [3,12,57]. The NOR contains ribosomal RNA (rRNA) genes and is the source for the formation of the nucleolus [82]. The nucleoli play numerous cellular functions, the prominent of which is rRNA synthesis, which gives rise to about 50% of total cellular RNA [83]. Within the nucleolus, the rDNA transcription occurs at the border of the dense fibrillar component (DFC) and fibrillar center (FC), while early processing of the nascent pre-rRNA transcript occurs in DFC [82]. Tau localises to the DFC [62] and the ribosomes [35,49]. Tau may not play a role in nucleolar assembly or formation [55], but together, these studies suggest it could be involved in rDNA transcription and/or rRNA processing (Figure 3). The synthesis of ribosomes begins within the nucleolus, with final maturation occurring in the cytoplasm. Hence, the localisation of tau both within the DFC of the nucleolus and to the ribosomes probably indicates that it may play a role in the processing of ribosomes from nascent pre-rRNA to maturation in the cytoplasm. Alternatively, nucleolar tau could play a part in the heterochromatinization of rDNA [62]. The majority of rDNA are kept in a transcriptionally inactive state through epigenetic mechanisms [84,85]. The silenced rRNA genes package to form a nucleolar heterochromatin localised to a region adjacent to the perinucleolar heterochromatin [86]. Sjöberg *et al.* showed that tau interacts with the perinucleolar heterochromatin and α-satellite of pericentromeric DNA [62]. This way, they proposed that tau could serve as a link between rDNA repeats and pericentromeric heterochromatin, through which it could play a role in rRNA gene silencing. Tau’s interaction with the perinucleolar heterochromatin makes it a potential regulator of rDNA stability, especially against illicit recombination [85,86]. In dividing cells, its association with the NOR of acrocentric chromosomes [3] also suggests a potential role for it in chromosomal stability [2]. Its role in chromosomal stability supported by the observation that cells with tau mutations showed a high degree of structural, numerical and stable chromosomal defects [2,87]. We are currently investigating the role of nucleolar tau in neuronal cells, but in-depth studies in dividing interphase non-neuronal cells are also warranted to completely understand its role in peripheral tissues and how it is influenced in diseases.

The capacity of tau to interact with nucleic acids, such as the RNA and DNA [68,69,70,71], and raise the melting temperature of the DNA suggests it could also play a role in DNA protection [72] (Figure 3). *In vitro* evidence showed that tau could protect the dsDNA from thermal denaturation and enhance its renaturation [78]. Hydroxyl free radical (–OH) are known to induce dsDNA breakage. *In vitro* evidence implicates tau in DNA protection from the –OH radical-induced DNA damage in a concentration-dependent manner [73,78,81,88]. How this DNA protective role occurs is not clear *in vivo*, especially because tau is mostly a cytosolic protein. It’s dynamic and reversible nature of interaction with the DNA [78] suggests that it may shuttle between the cytosol and the nucleus, similar to heat-shock proteins which shuttle between the cytoplasm and the nucleus following heat-shock [89,90,91]. Like tau, some of these heat-shock proteins are enriched in both cytoplasmic and nuclear compartments [92]. Heat-shock was previously shown to impact on tau protein phosphorylation in male and female rats [93]. Papasozomenos also found widely distributed tau immunoreactivity in the nucleus of an autopsy specimen of a subject with presenile dementia with motor neuron disease, cited in [57]. These findings led Papasozomenos to postulate that tau may play a role in stress response acting like heat-shock proteins (e.g., Hsp70) which upon heat shock translocate into the nucleus and to the nucleolus to maintain the integrity of the nuclear and nucleolar DNA and then subsequently exit to the cytoplasm [89,91,94]. Hsp70, which is known also to translocate to the nucleus upon heat shock, has been shown to protect the DNA [95]. In response to oxidative stress, Hsp72 was also shown to translocate to the nucleus, where it interacts with many nuclear proteins, including HMGB1, thus conferring cytoprotection by preventing the cytoplasmic translocation and release of HMGB1 from the injured cells [96]. In a somewhat similar scenario, tau has been shown to translocate to the nucleus following cellular distress to bind and protect the DNA in mouse primary neurons [52]. The immunogold electron microscopy image from Sultan *et al.*’s study [52] also showed dense nucleolar tau staining following the heat-stress, reminiscent of Hsp70 that translocates to the nucleus and the nucleolus to protect nucleoplasmic and ribosomal DNA from DNA breaks [94]. Similar to heat-shock proteins after the distress, Sultan *et al.* [52] showed that tau translocates back to the cytoplasm. Protein dephosphorylation has been previously shown to be essential for the translocation of Hsp70 into the nucleus in HeLa cells [90]. Interestingly, Sultan *et al.* showed that the nuclear tau under heat stress is mainly dephosphorylated [52]. Although, tau knockout (KO) mice are viable and show no apparent neuronal aberration [97], evidence from tau KO background also supports the DNA protective role of tau. Sultan *et al.* [52] showed that tau KO cortical neurons are vulnerable to heat stress-induced DNA damage while overexpression of hTau44 restored the DNA protective function of tau in this cells. Using wild-type and tau KO mice, the same group further showed a role for tau in RNA quality control and its reversible nuclear accumulation following cellular distress to protect the DNA [53]. To date, the role for tau in DNA protection is the only functional role of nuclear tau that has been clearly demonstrated both *in vitro* and *in vivo*. Generally, these findings and the similarities that exist between tau and heat-shock proteins reinforces the idea that tau could be a heat-shock protein. In our view, the tau that shuttles between the nucleus and the cytoplasm is a different isoform from the tau mainly localised to the nucleolus in neuronal and non-neuronal cell lines [3,62].

Furthermore, from the various studies [52,81], some features of the tau-DNA interaction is reminiscent of the interaction between chaperones and DNA [98]. An excellent example is that of HMGB1—a DNA chaperone and an architectural minor groove binder that binds the DNA without nucleotide sequence specificity. HMGB1 bends the DNA conformation and induces the formation of a multi-protein complex [99]. It stabilises the structure of the nucleosome, serves as a transcriptional facilitator and interacts with other minor groove binding proteins, such as TATA-binding protein (TBP) [96,100,101]. Interestingly, similar to HMGB1, tau protein also acts like a DNA-chaperone and architectural minor groove binder, as it binds the minor groove of DNA through its PRD and MBD without nucleotide sequence specificity thereby twisting the DNA, as well as changing the conformation of the DNA [52,80,102,103,104]. Tau also stabilises and prevents the denaturation of the DNA [73,81,88]. The literature revealed that minor groove architectural proteins can unwind the DNA, and also bend it so as to allow the formation of a multi-protein complex at the proximity of a specific region of the DNA [99,105]. This complex is formed by the recruitment of transcriptional factors that are bound at distantly separated DNA sites; in this way regulating gene expression [105]. Therefore, the minor groove binders enhance the assembly, stability and activity of the multiprotein-DNA complexes [99,105]. Previous studies also revealed that these proteins can stabilise the B-DNA conformation, and might function to reverse a curvature at a specific DNA site to create a structure that favours the recruitment of transcription factors. For instance, HMGI-Y was found to counteract on the intrinsic DNA curvature that exist on its binding site within the interferon β (INF-β) enhancer, thus allowing the recruitment of transcription factors by protein–protein interaction [106]. Furthermore, the bending of the DNA induced by these proteins can alter the conformation of the DNA at a distant site and thus affect the binding of other proteins at that site. And finally their nature of interaction with the DNA could also allow transcriptional inhibition [99]. Hence, minor groove binding proteins could have both transcriptional activation-like function and/or transcriptional inhibition-like function. They may not be direct transcriptional regulators, but they may indirectly affect the chromatin conformation and thus alter gene expression. This role has been well established for TATA-binding protein (TBP) which binds the minor groove of the TATA element of the DNA, kinking it, and thus leading to the formation of pre-initiation complex (PIC), which constitutes RNA polymerase II and other initiation factors (e.g., TFIIA) that alter transcriptional activity [105]. The formation of this multi-protein complex is heterogeneous, as it often involves interaction between different minor groove binding proteins (e.g., HMGB1-TBP interaction) [100]. Therefore, based on the current data on the tau–DNA interaction, it is not clear whether the primary function of tau is similar to that of the minor groove architectural proteins that solely bend the DNA to initiate the formation of multi-protein complex, or whether tau bends the DNA only as a way of protecting the DNA. Moreover, even though most transcription factors bind the DNA with sequence specificity, and typically via the major groove [99], but some also bind it without nucleotide sequence specificity, and such binding was proposed to be a way of finding its specific binding site [107]. We wonder whether this could explain the discrepancy in the literature where some studies showed that tau binds the DNA with sequence specificity [62,79], while others [78] showed that it binds the DNA non-specifically? Tau could function as a transcriptional regulator that first binds the DNA without sequence specificity, then later translocates to its correct DNA interacting sequence. With all the characters mentioned above of minor groove binding proteins, it appears attractive to speculate that tau protein being a minor groove binder could also function as a transcriptional activator by unwinding the DNA conformation. Indeed, Krylova *et al.* had earlier suggested that tau could be a transcriptional activator due to its ability to induce the separation of dsDNA to ssDNA [79]. Padmaraju *et al.* also showed that tau can alter gene expression by causing a change in DNA conformation from the standard B conformation to A–C conformations [103]. Qi *et al.* also showed that tau could bind GC-rich oligonucleotides [77]. CpG islands are found in GC-rich DNA regions and are essential regulators of transcription [108,109]. Some proteins with cytoplasmic localisation, such as Msn2, have also been shown to undergo dephosphorylation following cellular distress (e.g., Heat-shock) and translocate to the nucleus, where they function as a transcription factor [110,111]. Evidence from Tau KO mice showed that tau could regulate the *smarce1* gene, which encodes for BAF57 [112]. In an analysis of ~11,000 mRNAs using microarray screening from wild-type and tau KO mice, about 74 mRNAs were found to be significantly altered in the brain of 8-week-old KO mice. Further analysis using Q-RT-PCR, showed a significant rise in 13 mRNA in the KO mice brain [113]. These findings support a role for tau in nuclear gene regulation [97]. Therefore, a role for tau in transcriptional regulation is something worth investigating.

Generally, the understanding of the functional role of nuclear and nucleolar tau is preliminary; further studies are required *in vivo* to provide more details on tau’s nuclear role and interacting partners.

### 2.6. Role of Nuclear Tau in Neurodegeneration

Tau protein is known to be involved in the pathogenesis of many neurodegenerative diseases, especially due to its involvement in the regulation of neuronal microtubule and axonal transport. Hyperphosphorylation of tau, its aggregation to PHF and NFTs in vulnerable neurons is a hallmark of AD [5]. In AD, several reports indicate a reduction in NOR, rDNA transcription machinery and product, decreased protein synthesis machinery and impaired protein synthesis [104,114,115,116,117,118,119]. Nuclear tau has been localised in the AD brain, but compared to the control brain, no clear signature could be established that could indicate its role in the disease. This is probably because only a few cases were studied and variability could also exist due to the extent of pathology in the disease [50]. In AD, several reports indicate a reduction in NOR or rDNA transcription [104,114,115]. Tau localises to the nucleolus and NOR [3,12] and the ribosomes [35,49]. The nucleolar tau isNOR mainly dephosphorylated [3,12]. Upon phosphorylation, tau dissociates from the DNA [66]. If the nucleolar tau is involved in rRNA biogenesis, hyperphosphorylation during AD could dissociate it from the nucleolus, and, as a result, lead to altered ribosome biogenesis observed in AD. Interestingly, with the disease progression, a recent study reported a significant decrease in nuclear tau from many regions of the hippocampus and cortex, concomitant with significant aberration in the machinery regulating ribosome biogenesis, such as upstream binding factor (UBF), nucleolin (NLC) and nucleophosmin (NMP1) in some regions of the AD brain [119]. However, if nucleolar tau is involved with heterochromatinization of rDNA [62], it would remain unaltered or probably even more associated with the nucleolus, to promote the heterochromatinization of rDNA genes, which can also lead to the reduction of rDNA transcription. Indeed, the rDNA becomes hypermethylated in the mild cognitive impairment (MCI) and AD brain [120]. However, the depletion of nuclear tau with AD progression reported by Hernandez-Ortega *et al.* [119] does not appear to favour the later hypothesis. Moreover, the methylation strength of the rDNA promoter can also vary between brain regions and stage of the disease [120]. A detailed study is required to understand the role of tau in ribosome biogenesis, and for this, the brain region and disease severity must be taken into account to have a clear picture of its role.

Rossi *et al.* showed that tau in non-neuronal cells carrying the P301L tau mutation consistently present with a higher degree of structural, stable and numerical chromosome lesions, chromatin bridges and decondensed chromosomes [2]. This finding was furthered confirmed in other non-neuronal cells with varying tau mutations that rendered them more susceptible to genotoxic agents [87]. Considering the localisation of tau to the chromosomes [2,3,12], its capacity to bind and protect the DNA *in vitro* and *in vivo* [52,62,81] and the presence of different chromosomal aberrations and susceptibility to genotoxic stress in cells carrying tau mutation, tau was suggested to be essential for the maintenance of chromosome stability [87]. This suggests that the absence of normally functioning tau due to mutation can affect tau’s role in genome protection and render cells susceptible to chromosomal instability. Although tau’s potential role in chromosomal stability was demonstrated in non-neuronal cells [2,87], it does not preclude the potential of tau in stabilising the chromosome of neurons. Also, tau mutations do not cause AD, but nevertheless relationship exists between chromosomal instability and AD. For instance, an aberration in the *presenilin 1* (*PS-1*) gene has been shown to cause nondisjunction [121]. It was further demonstrated that familial Alzheimer’s disease (FAD) mutant APP (V717F) transgenic mouse and FAD-APP transfected cultured human cells, both produced chromosome missegregation and aneuploidy in both brains and peripheral cells [122]. These authors also showed that Aβ can also cause aneuploidy, including trisomy 21, in cultured cells. Interestingly, using spenocytes from tau KO mice, they further showed the direct involvement of tau in this chromosomal aberration induced by Aβ [122]. Increased level of aneuploidy specific to chromosome 21 has also been observed in the cerebral cortex of the AD brain [123]. Considering the role of tau in chromosomal stability [2,87], these studies could imply that in AD, increased production of Aβ could lead to tau aberration, such as phosphorylation, preventing it from protecting the genome, as well as stabilizing the chromosomes. Nuclear tau localises to chromosome 13, 14, 15, 21 and 22 [3]. Localisation of tau to these chromosomes has been earlier proposed to provide a link between AD and Downs syndrome (DS) (Trisomy 21) [3]. People with DS that live beyond 30 years develop AD later in life [124]. The connection could arise due to the overexpression of APP localised on chromosome 21, leading to increased Aβ production, which promotes chromosomal aberration [122] and can cause cellular dysfunction through both cytosolic and nuclear tau. Whether tau serves as the link between these two diseases and if aberration in tau contributes to the chromosome 21 specific aneuploidy observed by Ivan *et al.* [123] in the AD brain is something worth investigating.

It is also important to note that the involvement of nuclear tau in neurodegeneration could be through the stress-induced inhibition of normal tau function through hyperphosphorylation and aggregation [125]. Tau hyperphosphorylation and aggregation are among the principal modifications considered essential for the development of PHF and NFTs [5]. Hyperphosphorylation of tau reduces its nuclear translocation [126] and binding of tau to the DNA [76,77]. Aggregated tau also loses its ability to bind the DNA [75]. Hence, oxidative stress-induced tau hyperphosphorylation and aggregation could inhibit tau translocation to the nucleus and binding to protect the DNA [52,53,66,75]. However, this may not mean that phosphorylated tau completely becomes devoid of the nucleus. As indicated in previous sections, several reports showed that nuclear tau could be found in a phosphorylated state [2,50,56,66]. The existence of phosphorylated nuclear tau in normal cells is hard to explain, but it has been shown to exist in many pathological scenarios. For instance, the infection of human neuroblastoma SK-N-MC cells with Herpes simplex virus type 1 (HSV-1) led to the hyperphosphorylation and accumulation of tau in the nucleus [127]. Hyperphosphorylation of tau co-occurs with DNA damage in formaldehyde-treated N2a cells, indicating an involvement of the phosphorylated tau in DNA damage [73]. *In vitro* evidence suggests that phosphorylated tau can bind and alter the conformation and the integrity of the DNA, and in this way, could change nucleosomal organisation and impact on gene expression [103]. Indeed, recent findings from *Drosophila melanogaster* model of tauopathy, mouse model and evidence from human AD brain tissue revealed a central role of tau hyperphosphorylation in the induction of oxidative stress; DNA double-strand-breaks (DSBs); decompaction of the heterochromatin; aberrant gene dysregulation, especially of genes previously masked in the heterochromatin; cell cycle re-entry and neuronal apoptosis [65]. Although the involvement of nuclear tau in the induction of these changes has not been investigated in this study, tau phosphorylation was shown to be central to the induction of these pathogenic changes [65]. While writing this review, a new paper published by Hernandez-Ortega *et al*. [119] appears to provide an attractive link to the role of tau in the neurodegenerative process. The paper showed the depletion of nuclear tau in the neurons of the *cornus ammonis* 1 (CA1), dentate gyrus (DG), hilus, entorhinal cortex (EC) and temporal neocortex with AD progression. Nuclear tau becomes almost entirely depleted in NFT-bearing neurons of the CA1, EC and the temporal neocortex in later stages of the disease. Interestingly, these changes were associated with a reduction in chromatin marks involved with histone methylation (H3K9me3) and acetylation (H3k12ac) in the CA1 and DG neurons with disease progression. This finding suggests that tau modifications due to the disease stress cause the gradual depletion of tau from the nucleus even before NFTs formation and links this depletion to chromatin modifications. Increase in CSF phosphor-tau helps to predict the progression to AD from MCI [128]. Therefore, in AD, nuclear tau binding and protecting the DNA [52] or stabilizing the heterochromatin [62]; could be altered due to the change in the tau molecule configuration, such as phosphorylation, leading to its detachment from the DNA [73,77] and nuclear depletion [119], as a result, causing the alteration of chromatin integrity [65,103] and aberrant gene regulation. Future studies would provide a clear picture of the role of nuclear tau in neurodegeneration.

## 3. Conclusions

Since the discovery of tau in 1975, attention has focused largely on its microtubule-associated function and dysfunction in diseases. It is now clear that tau is a multifunctional protein. Its localisation to the nucleus in normal cells—phosphorylated and non-phosphorylated—is difficult to immediately understand, but its interaction with the DNA to protect the genome mainly when dephosphorylated supports the idea that aberrant modification in diseases like AD could alter its function and enhance genome vulnerability and neurodegeneration. Importantly, considering its wide distribution in the CNS and peripheral tissues, nuclear tau is potentially a global player for genome surveillance, in particular against chromosomal aberrations. Its interaction with other nuclear proteins strongly suggests it could play multiple roles in the nucleus, although further research is required to elucidate this. Our understanding of nuclear tau remains lacking, and to find efficient tau-based therapies, there is a need to understand more about the functional relevance of the varied cellular distribution of tau and how it is altered in the disease state.
